# Prediction of virus-host protein-protein interactions mediated by short linear motifs

**DOI:** 10.1186/s12859-017-1570-7

**Published:** 2017-03-09

**Authors:** Andrés Becerra, Victor A. Bucheli, Pedro A. Moreno

**Affiliations:** Escuela de ingeniería de sistemas y computación, Universidad del Valle, Calle 13 # 100-00, A. A. 25360, Cali, Colombia

**Keywords:** Virus, Host, Eukaryotes, Protein, Interaction, Prediction, Short, Linear, Motif, Disorder

## Abstract

**Background:**

Short linear motifs in host organisms proteins can be mimicked by viruses to create protein-protein interactions that disable or control metabolic pathways. Given that viral linear motif instances of host motif regular expressions can be found by chance, it is necessary to develop filtering methods of functional linear motifs. We conduct a systematic comparison of linear motifs filtering methods to develop a computational approach for predicting motif-mediated protein-protein interactions between human and the human immunodeficiency virus 1 (HIV-1).

**Results:**

We implemented three filtering methods to obtain linear motif sets: 1) conserved in viral proteins (*C*), 2) located in disordered regions (*D*) and 3) rare or scarce in a set of randomized viral sequences (*R*). The sets *C,D,R* are united and intersected. The resulting sets are compared by the number of protein-protein interactions correctly inferred with them – with experimental validation. The comparison is done with HIV-1 sequences and interactions from the National Institute of Allergy and Infectious Diseases (NIAID).

The number of correctly inferred interactions allows to rank the interactions by the sets used to deduce them: *D*∪*R* and *C*. The ordering of the sets is descending on the probability of capturing functional interactions.

With respect to HIV-1, the sets *C*∪*R*, *D*∪*R*, *C*∪*D*∪*R* infer all known interactions between HIV1 and human proteins mediated by linear motifs. We found that the majority of conserved linear motifs in the virus are located in disordered regions.

**Conclusion:**

We have developed a method for predicting protein-protein interactions mediated by linear motifs between HIV-1 and human proteins. The method only use protein sequences as inputs. We can extend the software developed to any other eukaryotic virus and host in order to find and rank candidate interactions. In future works we will use it to explore possible viral attack mechanisms based on linear motif mimicry.

**Electronic supplementary material:**

The online version of this article (doi:10.1186/s12859-017-1570-7) contains supplementary material, which is available to authorized users.

## Background

Virus-host Protein-protein interactions (VHPPIs) are essential to understand viral attack mechanisms. VHPPIs are used by viruses to disrupt or modulate host pathways in order to achieve goals like the evasion of the complement system [1], modulation of the cytokine system [2] and abrogation of apoptosis [3]. Some of these PPIs are based on mimicry: a viral protein mimicking a host protein might interact with the host protein binding partners. The mimicry is achieved through protein sequence or structural similarity [4]. We focus our study on predicting a subset of PPIs, the ones mediated by mimicked short linear motifs (SLiMs). SLiM-mediated PPI predictions, conveniently ranked, might help researchers to postulate hypothesis to elucidate viral attack mechanisms, design antivirals and vaccines [5–8].

A Short linear motif (SLiM) (also called linear motif, minimotif, ELM, LM) is a short region of a protein, 3 to 12 residues long, with functions like controlling the assembly of protein complexes, marking proteolytic cleavage, tagging protein localization and enzyme recruiting [9, 10]. SLiMs are structurally compact and participate in transitory low-affinity interactions [11, 12]. SLiMs in eukaryotic proteins are curated in the ELM database [13, 14].

SLiMs might evolve rapidly in viral disordered regions through insertions, deletions and mutations [15]. The new SLiMs can change the PPI networks creating new advantageous PPIs that can alter the cell cycle [16], form protein complexes and mediate conformational changes [17]. A recent analysis of the experimentally inferred human-virus PPIs concludes that human proteins interacting with viruses are enriched in SLiMs and binding interfaces [18].

Viruses use VHPPIs mediated by host-mimicked SLiMs to hijack cell regulation [19] and execute their viral cycle [20]. An example of this strategy is the set of SLiM-mediated interactions of human papilloma virus (HPV) protein E6 with members of the 14-3-3 protein family and proteins containing PDZ domains [21].

Experimental determination of VHPPIs is expensive since the number of proteins for some host organisms is large, more than 30.000 in humans. There are many viral protein sequences available but few corresponding three-dimensional structures resolved to use structure-based interaction prediction methods. These are reasons for developing a general method for predicting mimetic host-virus PPIs based solely in sequence data. A bioinformatic approach to predict SLiM-mediated VHPPIs might be an inexpensive alternative to experimentation or can guide experimental design.

SLiMs are represented computationally as regular expressions. A SLiM instance is a protein subsequence that matches the regular expression. For instance, a SLiM represented by the regular expression R.[RK]R. have several instances like RVRRE in Ebola virus [22] and RKRRF in Human respiratory syncytial virus A2 [23]. An algorithm for predicting virus-host PPIs consist in finding viral instances of SLiMs located in host proteins. The viral instances found need to be filtered by some criteria that increase the probability of inferring real interactions.

If a SLiM is conserved in a small viral genome it probably could be used to interact with a host protein. Evans et al. find that common SLiMs between HIV-1 and humans are significantly conserved in HIV-1 proteins [24]. They propose a criterion to filter SLiMs if they are conserved above a 70% in the available viral sequences.

Viral genomes have high mutation rates and are not too thermodynamically stable. This seems to favor protein structures with a small number of inter-residue interactions and a high number of polar residues that account for the abundance of disordered protein regions [25]. SLiMs occur more frequently in viral protein disordered regions [26], in different amounts between viral families [27]. Viral hubs, proteins that have many interactions with host proteins, tend to have more disordered regions [28]. With these antecedents Hagai et al. propose a criterion to filter SLiMs based on location in protein disordered regions [26].

Hagai et al. also propose another criterion to filter SLiMs based on rarity in a big set of randomized proteins [29]. A SLiM is judged as rare, or hard to form by pure chance, if it is counted in less than a percentage of the sequences in the set of randomized proteins, e.g. 1% of the sequences. Hagai et al. find that rare SLiMs located in disordered regions have a significant enrichment in functional SLiMs i.e. with experimental evidence for interaction with host proteins [29].

To our knowledge, there is no comparison of SLiM filtering methods in the literature. For that reason, we implement and compare the three criteria introduced above for SLiM filtering: conservation above a threshold of the available viral sequences, localization in a protein disordered region and rarity, or difficulty to form by pure chance. Each filtering method produces a set of SLiMs – conserved (*C*), disordered (*D*) and rare (*R*). With sets *C,D,R* we form derived union and intersection sets: *C*∪*D*, *C*∪*R*, *D*∪*R*, *C*∪*D*∪*R* and *C*∩*D*, *C*∩*R*, *D*∩*R*, *C*∩*R*∩*D*. Each of these sets allow us to predict interactions between the viral protein containing the SLiM and the host proteins that interact with the SLiM.

All the sets generated are compared by filtering strength. They also are compared by the number of VHPPIs derived from the set that have supporting evidence in a database –i.e. correctly predicted. The comparison by number of VHPPIs correctly predicted by set allow us to rank the VHPPIs partially.

To conduct the comparison of the sets we choose the Human immunodeficiency virus (HIV-1). It is the virus with more bioinformatic data available, with the NIAID databases for sequences and alignments [30] and for interactions with human proteins [31]. We also use the HIV-1-human PPIs mediated by SLiMs as reported in the LMPID database [32].

## Methods

### Disorder prediction

#### Protein preprocessing

We download alignments for HIV-1 proteins env, gag, nef, pol, rev, vif, vpr, tat, vpu for the year 2014 and an alignment of Gag-Pol DNA sequences with years previous to 2015 from the NIAID HIV-1 sequence database [30]. Gag-Pol sequences were translated following reference [33]: of 3648 sequences, 3626 containing the slippery subsequence TTTTTTA were used to perform a computational translation considering the frameshift at the given subsequence.

**Fig. 1:**
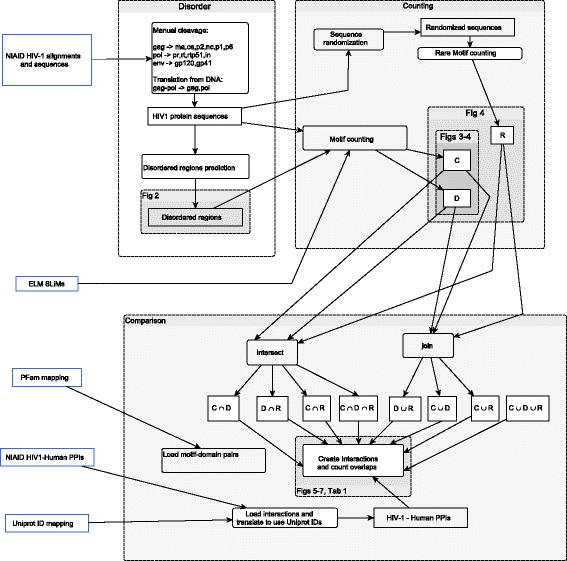
Methodology. The methods are *divided* in *three parts*: 1) Disorder: sequence preprocessing and prediction of disordered regions, 2) Counting: counting of SLiM *patterns* and instances, and 3) Comparison: analysis of the *overlap* between predicted interactions against interactions in NIAID and LMPID databases

We filter all protein sequences by HIV-1 subtypes B and C for their worldwide dominance and computationally cleave some of the alignments in the following manner: env into gp120, gp41, pol into pr, rt, rtp51, in and gag into ma, ca, p2, nc, p1, p6 [31]. After the cleavage we eliminate the gaps and asterisks in the resulting alignments in order to reinterpret the files as sets of sequences, Fig. 1, Disorder panel. The number of sequences per HIV-1 protein is in Additional file 1: Table S1.

#### Protein disorder prediction with IUPred

Among several disorder prediction algorithms for proteins [34] we use IUPred [35]. This predictor implements a physical model based on force fields between residues statistically calibrated with a set of globular proteins in PDB [35]. Its performance is comparable to other predictors [36] and can be installed locally.

IUPred is enhanced with a sliding window addition proposed by Hagai et al. that allows to define disordered regions [37]. Residues with IUPred computed values higher that 0.4 are considered disordered. For each residue an average disorder value is computed considering the IUPred values for surrounding residues in a window of size 10. This averaging is justified because the disorder tendency of the neighbors of a residue influence its disorder tendency. Residue windows with average disorder value higher than 0.4 are considered as disordered.

As IUPred receives as input a Fasta file with only one sequence, we split Fasta files with multiple sequences, call IUPred on every split sequence-file, compute the sliding window based average values and give as output a list of disordered regions per protein sequence id. We set the parameter *long* when calling IUPred, see Fig. 1, Disorder panel.

### Protein randomization

We randomize the HIV-1 proteins to create a big data set. For each sequence in a protein file we create 1000 shuffled versions randomizing the residues located in disordered regions of the sequence, as computed with IUPred. All disordered residues in a protein are joined together in a temporary list, shuffled with the modern Fisher–Yates algorithm, and put back in the disordered regions, leaving the ordered residues intact.

### SLiM counting

We download all the SLiMs, instances and interactions from the ELM database [14] and create an in-memory ELM data structure with each SLiM identifier, its regular expression, its instances and its interactions with protein domains. We wrote scripts to compute: the number of sequences with a given SLiM, the number of SLiM instances per protein, the number of SLiMs conserved above a percentage of sequences (set *C*) and the number of SLiMs in disordered regions (set *D*).

After randomizing as described above, we count the rare (scarce) SLiMs in these shuffled data set, i.e. the SLiMs that are found in 1% of the randomized sequences or less (set *R*).

Based on *C*, *D*, *R* we create the union sets *C*∪*D*, *C*∪*R*, *D*∪*R*, *C*∪*D*∪*R* and intersection sets *C*∩*D*, *C*∩*R*, *D*∩*R*, *C*∩*R*∩*D*. See Fig. 1, panels Counting and Comparison.

### Prediction of protein-protein interactions

We download the NIAID human–HIV-1 PPI database [31]. As the proteins in the database are identified by RefSeq records and the SLiM-domain interactions given by the ELM database are given by UniProt records, we map RefSeq to UniProt identifiers for human proteins using UniProt id mappings. We also download the LMPID database that curates virus-host ELM-mediated interactions [32].

For each SLiM set (*C,D,R*,…) obtained per HIV-1 protein we create VHPPIs based on the ELM database interactions and interacting domains. For each interaction reported in ELM we add the human protein interacting with the SLiM located in the viral protein. We also add the proteins that contain the domains listed as interacting with the SLiM. To map domains to human proteins we used the domain-protein mapping for the human proteome in the PFAM ftp server [38]. Figure 1, Comparison panel.

### Comparison of filtering methods

To validate a prediction we use two sets: the NIAID HIV-1-human interactions and the set of ELM mediated HIV-1-human interactions, as identified in LMPID [32]. We count the number of correctly predicted interactions, when an interaction deduced with one of the SLiM sets is in the NIAID database.

For all the SLiM sets obtained, and all the HIV-1 proteins, we analyze the overlap between the set of predicted human proteins interacting with HIV-1 and the set human proteins in NIAID interactions. We compute *p*-values for this overlap using the hyper-geometric distribution from the scipy python library, Table 1. The total number of human proteins was estimated as 30,057 from reference [39].

**Table 1:** *P*-values for the overlap between predicted interactions and NIAID PPIs

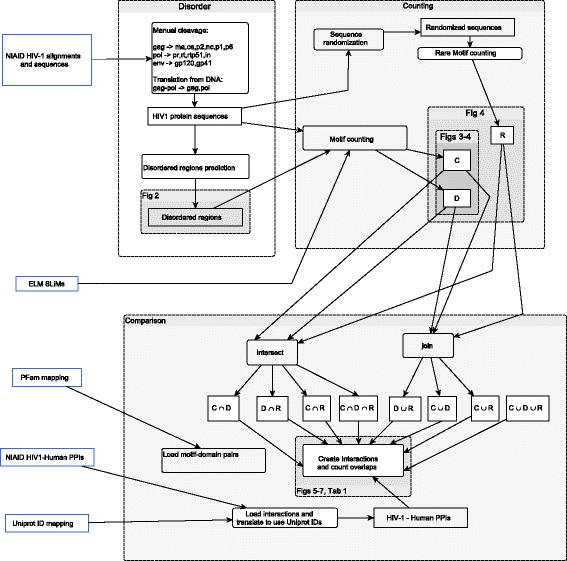									

The *p*-value indicates the probability that the overlap between our sets of predicted PPIs and the PPIs with literature support in the NIAID database takes place under the null hypothesis, that our sets were formed by random sampling. Red values are not significant at a level of 0.05

## Results and discussion

### A general method to identify SLiM-mediated PPIs in eukaryotes

As SLiMs are computationally represented by regular expressions there is always a possibility of finding instances in viral sequences by pure chance. For this reason, it is important to develop SLiM filtering methods.

Three filtering methods are implemented and systematically compared: conservation, location in disordered regions and rarity. The combination of filters produces a method to predict virus-host PPIs and rank them. The comparison of filtering methods performance is conducted with the virus with more abundant data, HIV-1. In Fig. 1 there is an overview of the methods used.

The developed method only use protein sequences as input and do not depend on protein 3D structures, for this reason it can be used with any sequenced eukaryotic virus to infer candidate VHPPIs. The restriction to eukaryotic viruses is based on the higher number of SLiMs in eukaryotes and the use of the ELM database, because the ELM SLiM classes MOD (post-translational modification) and TRG (targeting sites) are less used in prokaryotes [29].

#### Candidate interactions

The lists of predicted human-HIV-1 interactions that are not in the NIAID database are in the [Additional file 1].

### Disordered regions and SLiMs in HIV-1 proteins

**Fig. 2:**
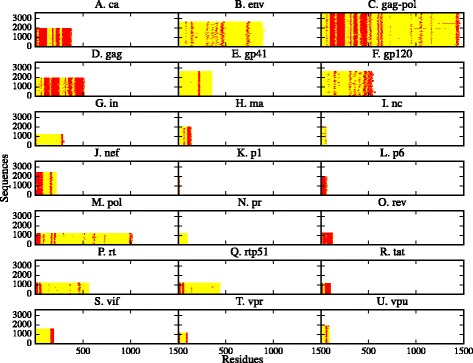
Disordered regions for HIV-1 proteins. Each subfigure from *A* to *U* contains an HIV-1 protein or protein precursor. For all subfigures, each *yellow line* represents a protein sequence. The *red* segments represent disordered regions as deduced with IUPred with the sliding window addition explained in the “Methods” section

The disordered regions for HIV-1 proteins are in the [Additional file 2: Table S2]. They are depicted in Fig. 2. Subfigures A to U show the predicted disordered content in HIV-1 proteins and polyproteins. Each protein sequence is represented as a yellow line and disordered regions are depicted as red segments.

We find that predicted disordered regions for HIV-1 proteins are relatively conserved. Perhaps the virus must keep flexibility in their proteins in order to interact with several partners.

**Fig. 3:**
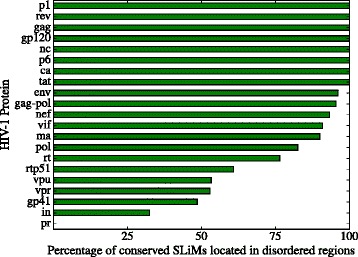
Percentage of conserved SLiMs that are located in disordered regions in HIV-1. We *plot* the percentage of conserved SLiMs, present in 70% or more of the input sequences, that are localized in a predicted disordered region

In Fig. 3, we show the percentage of SLiMs conserved above a 70% of the input sequences that are also located inside a disordered region. Most of the conserved SLiMs in HIV-1 are located in protein disordered regions.

The proteins that deviate the most from this tendency are vpr, vpu, gp41, in, and pr, with a percentage of conserved motifs that are located in disordered regions of 53.3, 52.9, 48.5, 32.5 and 0% respectively. The reason for this discrepancy lies in the few disordered regions predicted in the five proteins. Indeed, pr, in and gp41 are considered mostly ordered, while vpr and vpu are considered moderately disordered [40].

A similar correlation between evolutionary conservation and location in disordered regions was found for the SLiMs that bind to SH2, SH3 and Ser/Thr Kinase domains [41].

We use IUPred as disorder predictor only because its performance finding the disordered regions of the VIF protein is outstanding compared to other 18 disorder predictors [36]. One procedure that could be used to avoid structured regions entirely is a BLAST query against HIV-1 proteins in the Protein Data Bank excluding hit regions. However, it seems that disorder is a viral strategy to buffer mutations and increase interactions with host proteins [42]. In this perspective, small disordered regions could be located inside structured protein regions to allow some interactions with the host, and not excluding the structured regions opens the possibility of finding these regions.

### Analysis of SLiM sets obtained

#### A ranking of SLiM sets by filtering strength

**Fig. 4:**
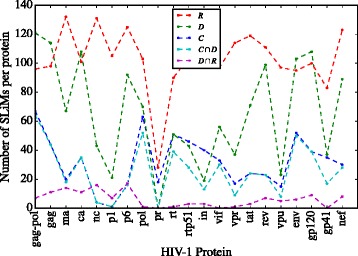
Number of SLiMs by set. We *plot* the number of SLiMs (regular expressions) that were found in HIV-1 proteins. The intersection SLiM sets *C*∩*R* (conserved and rare) and *C*∩*D*∩*R* (conserved, rare, located in disordered regions) were discarded for being almost null in all entries

The SLiM set sizes are in Additional file 3: Table S3 and the SLiM sets for HIV-1 proteins are in the [Additional file 3]. In Fig. 4 we plot the the number of SLiM regular expressions that were found in the HIV-1 proteins identified by set. The intersection SLiM sets *C*∩*R* (conserved and rare) and *C*∩*D*∩*R* (conserved, rare, located in disordered regions) were discarded for being almost empty for all proteins.

Considering the sizes of SLiM sets we can rank them by the filtering strength; from low to high filtering. The obtained ranking is *R,D,C,C*∩*D,D*∩*R*. The criterion that filters the most is location in a disordered region and rarity. It is followed by location in a disordered region and conservation.

The sets *D*∩*R* (SLiMs hard to form by pure chance and located in protein disordered regions) studied by Hagai et al. [29], tend to have a smaller size than sets *C*∩*D*, of SLiMs conserved and located in protein disordered regions, Fig. 4. The intersection SLiM sets *C*∩*R* (conserved and rare) and *C*∩*D*∩*R* (conserved, rare, located in disordered regions) are almost empty so they can be discarded as useful filtering criteria –data in Additional file 3: Table S3.

### Protein-protein interactions predicted with the SLiM sets are enriched in experimentally validated HIV-1-human protein-protein interactions

We validate against two virus-host PPIs databases: NIAID [31] and LMPID [32]. The NIAID contains 15074 PPIs at the moment of writing while LMPID contains 2203 PPIs between several viruses and hosts, with 6 PPIs between HIV-1 and human proteins.

The validation of the predicted PPIs with the NIAID database is not the best way to gauge the proportion of SLiM-based interactions. This database contains PPIs of all kinds, not only SLiM-mediated ones. However, it is the most complete virus-host PPI dataset.

A better validation set, conceptually, is constructed with pairs deemed to interact through a SLiM with the LMPID database. Nevertheless, this dataset is too small. We do the comparison with both databases, selecting the NIAID database to compare the sets prediction performance and check the statistical significance of the results.

Although we are suggesting a partial ranking of SLiM-based predicted PPIs, another addition would be to rank totally the interactions with a score representing the probability that the interaction takes place based on experimental data [43] or other techniques [44]. For the moment, a total ranking is difficult to achieve given the scarcity of data about SLiM-mediated PPIs [32, 45].

#### In the NIAID database

**Fig. 5:**
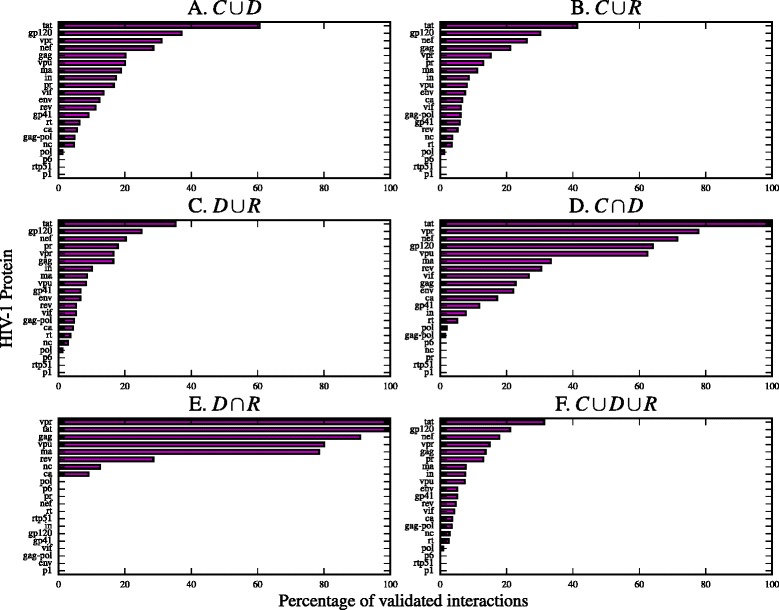
Percentage of validated interactions per SLiM set. Each subfigure *plots* the percentage of validated interactions with a SLiM set. Interactions were validated with the NIAID HIV-1 Human Interaction Database. The percentage of predicted interactions is represented with a *magenta bar*. The HIV-1 proteins are sorted by percentage in each subfigure

**Fig. 6:**
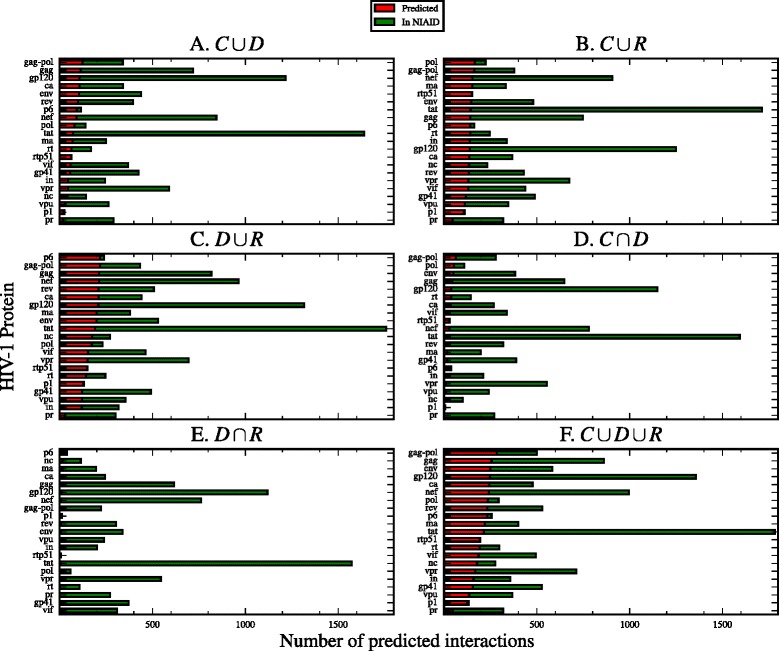
Number of predicted interactions per SLiM set. The number of predicted interactions by SLiM set, contrasted with the number of interactions in the NIAID HIV-1 Human Interaction Database. The number of predicted interactions is represented with a *red bar*. The *green bar* represents the total number of interactions in the NIAID database

In Fig. 5 we plot the percentage of correctly predicted interactions, i.e. stored in the NIAID database and predicted with base on our SLiM sets. In Fig. 6 we plot the number of interactions predicted against the total number of interactions in the NIAID database per HIV-1 protein. The number of correctly predicted interactions is in Additional file 4: Table S4 and the number of novel interactions found with our method is in Additional file 4: Table S5.

We use the hyper-geometric distribution to measure the statistical significance of the sets of interactions we found. The *p*-values for the overlap between the PPIs predicted with base on each SLiM set and the PPIs in the NIAID database are in Table 1. The sensitivity and specificity for the SLiM sets as PPI predictors is in Additional file 4: Table S7 and Additional file 4: Table S8.

#### In the LMPID database

Using the literature curated LMPID database [32], we find that the motif sets *C,C*∩*D,C*∩*R,C*∩*D*∩*R* capture half of the interactions in LMPID, while the sets *C*∪*D,C*∪*R,D*∪*R,C*∪*D*∪*R,D*∪*R* allow to infer all of them. All the interactions between HIV-1 and human extracted from LMPID are in Additional file 4: Table S6.

The small number of human-HIV-1 interactions in this database (six), leaves open two possibilities: the number is really small, or the number is larger but few experiments have been performed to detect them. To estimate the number of human-HIV-1 SLiM-mediated PPIs more work is needed, perhaps an approach based on combining expert opinions [46].

#### PPIs correctly predicted serve as a ranking of filtering methods

**Fig. 7:**
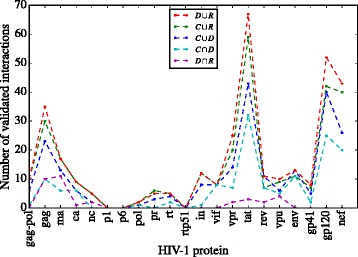
Number of validated interactions per set. The number of interactions as validated with the NIAID HIV-1 Human Interaction Database. Each set is represented by a *dashed line* with a different *color*

In Fig. 7 we plot the number of predicted interactions correctly validated against the NIAID database identified by the SLiM set used to infer them. We find that the SLiM sets have an almost general tendency with respect to the number of PPIs correctly predicted across all HIV-1 proteins. For this reason we propose to rank the PPIs predicted according to the set used to deduce them.

The ranking of the sets we found by its capacity to infer real interactions is: *D*∪*R,C*∪*R,C*∪*D,C*∩*D,D*∩*R*. This ranking allow to present the PPIs predicted to researchers in a partial order: first the set of interactions deduced with *D*∪*R* –SLiMs located in disordered regions or hard to form by pure chance, then the set deduced with *C* –conserved SLiMs.

#### Most used SLiMs in HIV-1 proteins suggest HIV-1 extensive use of human protein signaling and other processes

We consider the set *C*∩*D* of SLiMs conserved and located in disordered regions for their biological relevance to analyze their human counter domains. In Table 2 we include the most used SLiMs from this set, i.e the SLiMs that are present in 10 or more HIV-1 proteins, are conserved, and localize in a disordered region. In general, most of these SLiMs would interfere with host signaling. The most used counter domains are the Protein kinase domain (PF00069 in Pfam) that interact with 5 of the most used SLiMs and the Peptidase_S8 Subtilase family (PF00082 in Pfam) that interacts with 2 of the most used cleavage SLiMs. However, the list of counter domains in Table 2 suggest that HIV-1 SLiMs interfere with transcription regulation, autophagy, cell cycle control, apoptosis and cellular transport.

**Table 2:** Most used SLiMs conserved and located in HIV-1 disordered regions

SLiM	#HIV-proteins	Pfam domain	Domain name
LIG_WD40_WDR5_VDV_2	17	IPR017986	WD40-repeat-containing domain
DOC_USP7_1	17	PF00917	MATH domain
CLV_NRD_NRD_1	16	PF00675	Insulinase (Peptidase family M16)
CLV_PCSK_KEX2_1	15	PF00082	Peptidase_S8 Subtilase family
MOD_GSK3_1	15	PF00069	Protein kinase domain
MOD_PIKK_1	15	PF00454	Phosphatidylinositol 3- and 4-kinase
CLV_PCSK_SKI1_1	14	PF00082	Peptidase_S8 Subtilase family
LIG_SH3_3	14	PF00018	SH3 domain
MOD_NEK2_1	13	PF00069	Protein kinase domain
LIG_FHA_2	13	PF00498	FHA domain
MOD_CK1_1	13	PF00069	Protein kinase domain
LIG_FHA_1	12	PF00498	FHA domain
DOC_CYCLIN_1	12	PF00134	Dynein light chain type 1
LIG_LIR_Nem_3	12	PF02991	Autophagy protein Atg8 ubiquitin like
DOC_WW_Pin1_4	12	PF00397	WW domain
MOD_ProDKin_1	12	PF00069	Protein kinase domain
MOD_PKA_2	12	PF00069	Protein kinase domain
MOD_CK2_1	12	PF00069	Protein kinase domain
TRG_ER_diArg_1	12	PF00400	WD domain, G-beta repeat
LIG_SH2_STAT5	11	PF00017	SH2 domain
LIG_LIR_Gen_1	11	PF02991	Autophagy protein Atg8 ubiquitin like
CLV_PCSK_PC1ET2_1	11	PF00082	Peptidase_S8 Subtilase family
MOD_GlcNHglycan	11	PF01048	Phosphorylase superfamily
MOD_N-GLC_1	10	PF02516	Oligosaccharyl transferase STT3 subunit
TRG_ENDOCYTIC_2	10	PF00928	Adaptor complexes medium subunit family

From the SLiMs that are conserved in more than 70% of the HIV-1 protein sequences, and are located in disordered regions too we counted how many HIV-1 proteins include them. In this table we report the SLiMs more commonly used in HIV-1 proteins, the ones that are included in 10 or more of the HIV-1 proteins. The table includes the counter domain for every SLiM

## Conclusion

We develop a method to predict virus-host SLiM-mediated PPIs and rank them. It is applicable to any eukaryotic virus and host with available protein sequences. Using data for the most studied virus, HIV-1, we find a partial ordering of the PPIs obtained based on the set used to infer the interactions. This order is descending in the expected probability of inferring real interactions. We expect that the method gives interesting candidate interactions with other eukaryotic viruses and hosts. The call for using high-throughput methods to detect SLiM-mediated PPIs illustrates the benefits of a bioinformatic method that predicts SLiM-mediated PPIs and might guide experimental design [45].Although the number of SLiM-mediated PPIs might be small, there is evidence that these PPIs are used by several viruses, in contrast to virus-host domain-domain PPIs, that are virus-specific [18]. This kind of interactions can help to analyze common viral strategies for infection.

Indeed, in a previous work we used the method with the viruses in the NCBI virus variation resource to predict interactions with the proteins from the human protein synthesis machinery [47]. We found evidence that viruses interact with Eukaryotic initiation factors 3 and 4, and the Poly(A)-binding proteins using SLiMs. Even though the method developed is not a strong predictor, by using several viruses, interesting interactions with host subsystems can be uncovered. In a future work we want to scale the approach considering all the human proteome and more human viruses.

In future work we could also incorporate structural information in the prediction and analysis of SLiM-mediated VHPPIs in order to create otter SLiM filtering methods and compare them with the filters obtained in this work. One possibility is the study of fuzziness and SLiM flanking regions [48], another one is the use of disordered binding region prediction methods, like ANCHOR [49].

## Additional files

Additional file 1
**Table S1.** Candidate interactions between human and HIV-1 (interactions.zip) available at https://figshare.com/articles/interactions_zip/4648714. (ZIP 2549.76 kb)

Additional file 2
**Table S2.** Disordered regions for HIV-1 proteins (regions.zip), using filenames layout explained in Table S2 available at https://figshare.com/articles/Disordered_regions_predicted_in_HIV-1/4648729. (ZIP 708 kb)

Additional file 3
**Table S3.** SLiM sets *C,D,R* and derived for HIV-1 proteins. (SLiM_sets.zip) available at https://figshare.com/articles/Short_Linear_Motif_Sets_common_to_human_and_HIV-1/4648732. (ZIP 145 kb)

Additional file 4
**Table S4.** Supplement information describing the previous files. (Supplement - Prediction of Virus-Host Protein-Protein interactions based on Short Linear Motifs.pdf) available at https://figshare.com/articles/Supplement_-_Prediction_of_Virus-Host_Protein-Protein_interactions_based_on_Short_Linear_Motifs/4667461. (PDF 166 kb)

## Abbreviations

ELM: Eukaryotic linear motifs
HIV-1: Human immunodeficiency virus - 1
LMPID: Linear motif mediated protein interaction database
NIAID: National institute of allergy and infectious diseases
PDB: Protein data bank
Pfam: Protein families database
PPI: Protein-protein interaction
SLiM: Short linear motif
VHPPI: Virus-host protein-protein interaction
