# Supplementary material for: Prediction of virus-host protein-protein interactions mediated by short linear motifs
Source: BMC Bioinformatics. 2017 Mar 9;18:163. doi: 10.1186/s12859-017-1570-7 (PMC5345135; doi:10.1186/s12859-017-1570-7)
Supplement: Additional file 4 — Table S4. Supplement information describing the previous files. (Supplement - Prediction of Virus-Host Protein-Protein interactions based on Short Linear Motifs.pdf) available at https://figshare.com/articles/Supplement_-_Prediction_of_Virus-Host_Protein-Protein_interactions_based_on_Short_Linear_Motifs/4667461. (PDF 166 kb) [file 12859_2017_1570_MOESM4_ESM.pdf]

Supplemental information Prediction of virus-host protein-protein interactions based on short linear motifs

Andrés Becerra, Victor A. Bucheli, Pedro A. Moreno
{andres.becerra,victor.bucheli,pedro.moreno}@correounivalle.edu.co

Full list of author information is available at the end of the article

S1 HIV-1 Sequences

The number of sequences obtained from the NIAID database is given in Table S1 [1]. The number of randomized sequences generated to obtain the rare motif set (R) are 1000 times the values presented in Table S1. The randomized sequences are available on request.

Table S1 Number of sequences per HIV-1 protein

Protein	Sequences
ca	1942
env	2600
gag-pol	3626
gp41	2600
gp120	2600
in	1182
ma	1942
nc	1942
nef	2415
p1	1942
p6	1942
pol	1182
pr	1182
rev	1239
rt	1182
rtp51	1182
tat	1131
vif	1517
vpr	1113
vpu	1873

S2 Disordered regions

The disordered regions were computed using IUPred [2] with the window addition explained in the methods section [3]. The results are in in text files named with the pattern regions-proteinName, where proteinName is one of the protein names in Table S1.

The files have the following layout:

```
B.FR.83.HXB2_LAI_IIIB_BRU.K03455716-1004,   275 287  ,
B.AR.00.ARMS008.AY037269716-1004,   274 287  ,
B.AR.03.03AR137681.DQ383748716-1004,   274 287  ,
B.AR.03.03AR138910.DQ383749719-1007,   274 287  ,
B.AR.98.ARCH054.AY037268716-1004,   274 287  ,
```

Each line contains a sequence id and a list of comma-separated disordered regions. Each disordered region is represented as a pair of sequence coordinates separated by a space. The predicted regions per protein are in the [Additional file 2].

S3 Short Linear Motifs

The SLiM sets computed were generated as CSV files with names like ca_motifs_D.csv with the HIV-1 protein name as prefix, and the set name as suffix. The protein name is one of the names in Table S1, the suffixes used are in Table S2. Unions like $C \cup D$ were represented in filenames as suffixes `uCD` and intersections like $D \cap R$ as `iDR`.

Each SLiM is identified in the files by the same identifier used in the ELM database (e.g. LIG_GSK3_LRP6_1) [4, 5]. The number of SLiMs per set is reported in Table S3 and the actual SLiMs are in the [Additional file 3].

Table S2 Suffixes used in the filenames of the the SLiM sets

SLiM set	Suffix
Conserved [6]	C
Disordered [7]	D
Rare [7]	R
Conserved and disordered	iCD
Conserved and rare	iCR
Disodered and rare	iDR
Conserved and disordered and rare	iCDR
Conserved or disordered	uCD
Conserved or rare	uCR
Disodered or rare	uDR
Conserved or disordered or rare	uCDR

Table S3 Number of SLiMs by filter criterion.

Protein	$\|C\|$	$\|D\|$	$\|R\|$	$\|C \cup D\|$	$\|C \cup R\|$	$\|D \cup R\|$	$\|C \cup D \cup R\|$	$\|C \cap D\|$	$\|D \cap R\|$
ca	35	108	101	108	136	209	244	35	11
env	52	103	95	105	147	198	250	50	6
gag	44	114	98	114	142	212	256	44	11
gp41	35	37	83	55	118	120	155	17	0
gp120	39	108	100	108	139	208	247	39	9
in	40	19	100	46	140	119	159	13	3
ma	20	67	132	69	152	199	219	18	14
nc	4	43	131	43	135	174	178	4	16
nef	30	89	123	91	153	212	242	28	8
p1	1	21	105	21	106	126	127	1	7
p6	16	92	125	92	141	217	233	16	17
pol	63	70	103	81	166	173	236	52	1
pr	18	0	28	18	46	28	46	0	0
rev	23	99	111	99	134	210	233	23	7
rt	51	51	90	63	141	141	192	39	1
rtp51	46	43	104	61	150	147	193	28	3
tat	24	71	119	71	143	190	214	24	3
vif	33	56	97	59	130	153	186	30	0
vpr	17	37	114	45	131	151	168	9	1
vpu	15	23	97	30	112	120	135	8	5

For HIV-1 proteins we report the number of SLiMs conserved in more than 70% of the HIV-1 sequences (C), the number of SLiMs found in disordered protein regions (D), the number of rare –hard to form by pure chance– SLiMs (R) and the derived union and intersection sets sizes. Column values for sets $C \cap R$ (conserved and rare) and $C \cap D \cap R$ (conserved, rare, located in disordered regions) are not included for being almost null.

S4 Interactions

S4.1 Human-HIV1 interactions in the NIAID database

The number of predicted interactions per set and HIV-1 protein is presented in Tables S4 and S5. The first one presents the interactions validated in the NIAID database and the second one the interactions not present in the NIAID database [8], the candidate interactions. These interactions are in the [Additional file 1], this is a CSV file named with the pattern: `hivProtein-interactions-suffix.csv`.

Where hivProtein is one of the names in Table S1 and the suffix is one of the names in Table S2. Each file includes the human proteins using their Uniprot id. Each file contains for every HIV-1 protein, and SLiM set used for inference, a set of human proteins identified by Uniprot ids [9].

Table S4 Number of predicted interactions based on the SLiM set that have experimental support in NIAID PPI database.

	C	D	R	$C \cup D$	$C \cup R$	$D \cup R$	$C \cup D \cup R$	$C \cap D$	$D \cap R$
ca	6	6	6	6	9	9	9	6	1
env	11	13	6	13	11	13	13	11	0
gag-pol	0	6	10	6	10	10	10	0	5
gag	10	23	27	23	30	35	35	10	10
gp41	4	3	7	5	7	8	8	2	0
gp120	25	40	37	40	42	52	52	25	0
in	8	1	12	8	12	12	12	1	0
ma	6	13	16	13	17	17	17	6	11
nc	0	2	5	2	5	5	5	0	2
nef	20	26	34	26	40	43	43	20	0
p1	0	0	0	0	0	0	0	0	0
p6	0	0	0	0	0	0	0	0	0
pol	1	1	2	1	2	2	2	1	0
pr	3	0	5	3	6	5	6	0	0
rev	7	11	7	11	7	11	11	7	2
rt	4	2	5	4	5	5	5	2	0
rtp51	0	0	0	0	0	0	0	0	0
tat	32	43	53	43	59	67	67	32	3
vif	8	8	6	8	8	8	8	8	0
vpr	7	14	20	14	20	25	25	7	2
vpu	5	6	9	6	9	10	10	5	4

Table S5 Interactions predicted and not validated in the NIAID database

protein	C	D	R	$C \cup D$	$C \cup R$	$D \cup R$	$C \cup D \cup R$	$C \cap D$	$D \cap R$
ca	3045	6483	5743	6483	7319	9008	9008	3045	1027
env	4382	5999	5648	6033	7730	9028	9061	4348	598
gag-pol	5255	7179	5139	7326	8192	9339	9405	5108	199
gag	4237	6794	5386	6794	7614	9139	9139	4237	1030
gp41	3735	4067	5208	4851	6617	6908	7497	2751	0
gp120	3819	6058	5419	6058	7100	8832	8832	3819	678
in	3828	1613	6323	3915	7606	6605	7645	1476	62
ma	2922	5134	7007	5175	7955	8752	8760	2692	2654
nc	194	4462	7533	4462	7578	8146	8146	194	2971
nef	3375	5793	6875	5826	8053	9672	9691	3341	1017
p1	0	2771	6967	2771	6967	7497	7497	0	1417
p6	2008	5492	6910	5492	7552	9365	9365	2008	1465
pol	4854	5354	5917	5703	8377	8840	9025	4498	140
pr	2744	0	2937	2744	4069	2937	4069	0	0
rev	3282	6340	6143	6340	7052	9044	9044	3282	935
rt	4075	4288	5638	4985	7762	7753	8126	3366	50
rtp51	4115	4003	6482	5220	8269	8274	8772	2825	113
tat	2753	4928	6545	4928	7259	8932	8932	2753	159
vif	3284	4026	5583	4075	6766	7414	7442	3215	0
vpr	2312	3381	6922	3920	7696	7989	8491	1723	96
vpu	2420	2916	5769	3353	6375	6426	6690	1895	1439

The number of interactions predicted is high in many cases. The reason is the number of proteins and isoforms that contain a domain that is deemed to interact with a SLiM.

S4.2 Human-HIV1 interactions in the LMPID database

In Table S6 we report the SLiM-mediated interactions between HIV-1 and humans that were extracted from the LMPID database [10].

Table S6 Interactions between HIV-1 and human proteins mediated by a SLiM as identified in LMPID.

**HIV-1**	**Human Protein Name**
gag	Programmed cell death 6-interacting protein
gag	Tumor susceptibility gene 101 protein
nef	Tyrosine-protein kinase HCK
nef	Tyrosine-protein kinase Fyn
tat	Serine/threonine-protein phosphatase PP1-alpha catalytic subunit
vpu	F-box/WD repeat-containing protein 1A

S5 Sensitivity and specificity

Although there is no gold-standard dataset for VHPPIs we use the NIAID database to estimate the sensitivity of the SLiM-based predictions. We iterate through all possible interactions between human and HIV-1 proteins to compute the true positives, true negatives, false positives and false negatives. Tables S7 and S8 report the sensitivity and specificity. The values are discriminated per HIV-1 protein and SLiM set used to infer the intearcions.

S6 Mappings used

S6.1 RefSegs to Uniprot Ids

To map the NIAID human-HIV-1 interactions given in RefSeqs to Uniprot Ids given in the ELM database we use the files in the Uniprot FTP. contains the correspondence between Uniprot ids and other databases ids like RefSeq.

`ftp://ftp.uniprot.org/pub/databases/uniprot/current_release/knowledgebase/idmapping/by_organism/HUMAN_9606_idmapping.dat.gz`

Table S7 Sensitivity percentage for SLiM sets prediction over HIV-1 proteins

protein	C	D	R	$C \cup D$	$C \cup R$	$D \cup R$	$C \cup D \cup R$	$C \cap D$	$D \cap R$
ca	1.99	3.90	3.82	3.90	4.54	5.16	5.16	1.99	0.75
env	2.12	2.97	3.13	2.97	3.79	4.42	4.42	2.12	0.19
gag-pol	3.67	4.05	3.07	4.45	4.82	5.03	5.14	3.27	0.09
gag	2.21	3.16	2.26	3.16	3.31	3.83	3.83	2.21	0.50
gp41	2.30	2.58	3.03	2.97	3.70	3.91	4.13	1.81	0.00
gp120	1.17	1.85	1.84	1.85	2.12	2.60	2.60	1.17	0.23
in	2.56	1.72	3.86	2.65	4.45	4.28	4.48	1.63	0.08
ma	1.74	2.78	3.77	2.83	4.21	4.57	4.57	1.35	1.36
nc	0.18	3.00	4.24	3.00	4.24	4.60	4.60	0.18	2.33
nef	1.14	2.31	2.49	2.31	2.62	3.28	3.28	1.14	0.41
p1	0.00	2.04	4.55	2.04	4.55	4.82	4.82	0.00	1.53
p6	1.52	3.36	4.84	3.36	5.07	5.80	5.80	1.52	1.17
pol	3.59	3.43	4.03	3.88	5.57	5.61	5.76	3.14	0.01
pr	1.48	0.00	2.02	1.48	2.57	2.02	2.57	0.00	0.00
rev	2.08	3.79	3.99	3.79	4.44	5.27	5.27	2.08	0.21
rt	2.75	2.60	3.46	3.01	4.54	4.51	4.69	2.34	0.00
rtp51	3.09	2.64	4.41	3.60	5.41	5.38	5.63	2.11	0.09
tat	0.98	1.48	2.17	1.48	2.39	2.73	2.73	0.98	0.05
vif	2.01	2.42	3.22	2.42	3.90	4.19	4.19	1.94	0.00
vpr	0.81	1.65	3.11	1.86	3.36	3.59	3.81	0.59	0.06
vpu	1.34	1.72	2.75	1.95	2.89	3.28	3.34	0.93	1.00

Table S8 Specificity percentage for SLiM sets prediction over HIV-1 proteins

protein	C	D	R	$C \cup D$	$C \cup R$	$D \cup R$	$C \cup D \cup R$	$C \cap D$	$D \cap R$
ca	98.87	97.62	97.4	97.62	96.91	96.41	96.41	98.87	99.61
env	98.40	97.74	97.41	97.72	96.84	96.41	96.40	98.42	99.71
gag-pol	97.98	97.38	97.6	97.28	96.59	96.36	96.31	98.08	99.94
gag	98.41	97.57	97.56	97.57	96.86	96.43	96.43	98.41	99.61
gp41	98.58	98.34	97.85	98.09	97.47	97.32	97.15	98.91	100.00
gp120	98.65	97.77	97.62	97.77	97.19	96.6	96.60	98.65	99.69
in	98.53	99.3	97.48	98.51	97.09	97.36	97.09	99.33	99.97
ma	98.84	98.08	97.19	98.06	96.91	96.74	96.73	98.96	98.79
nc	99.91	98.25	97.09	98.25	97.07	96.91	96.91	99.91	98.79
nef	98.72	97.87	97.06	97.87	96.74	96.25	96.25	98.72	99.59
p1	100.00	98.9	97.18	98.9	97.18	97.00	97.00	100.00	99.40
p6	99.24	97.96	96.97	97.96	96.85	96.33	96.33	99.24	99.37
pol	98.13	97.94	97.35	97.76	96.59	96.44	96.36	98.31	99.90
pr	98.93	100.00	98.86	98.93	98.48	98.86	98.48	100.00	100.00
rev	98.60	97.65	97.23	97.65	96.9	96.44	96.44	98.60	99.58
rt	98.46	98.41	97.47	98.06	96.82	96.89	96.69	98.80	99.99
rtp51	98.38	98.48	97.16	97.95	96.59	96.63	96.42	98.96	99.98
tat	98.87	98.18	97.28	98.18	97.06	96.55	96.55	98.87	99.95
vif	98.74	98.52	97.52	98.50	97.20	96.99	96.99	98.76	100.00
vpr	99.09	98.71	97.04	98.53	96.8	96.75	96.58	99.27	99.95
vpu	99.03	98.81	97.5	98.66	97.34	97.32	97.25	99.23	99.37

S6.2 Domains to Proteins

To map the domains given in the ELM database to proteins containing them we use the PFam mapping from the ftp:

`ftp://ftp.ebi.ac.uk/pub/databases/Pfam/releases/Pfam28.0/proteomes/9606.tsv.gz.`

References

1. Kuiken C, Korber B, Shafer RW. HIV sequence databases. AIDS Rev. 2003;5(1):52–61.
2. Dosztanyi Z, Csizmok V, Tompa P, Simon I. The pairwise energy content estimated from amino acid composition discriminates between folded and intrinsically unstructured proteins. J Mol Biol. 2005 Apr;347(4):827–839.
3. Hagai T, Azia A, Toth-Petroczy A, Levy Y. Intrinsic disorder in ubiquitination substrates. J Mol Biol. 2011 Sep;412(3):319–324.
4. Dinkel H, Michael S, Weatheritt RJ, Davey NE, Van Roey K, Altenberg B, et al. ELM–the database of eukaryotic linear motifs. Nucleic Acids Res. 2012 Jan;40(Database issue):D242–251.
5. Dinkel H, Van Roey K, Michael S, Kumar M, Uyar B, Altenberg B, et al. ELM 2016-data update and new functionality of the eukaryotic linear motif resource. Nucleic Acids Res. 2015 Nov;.
6. Evans P, Dampier W, Ungar L, Tozeren A. Prediction of HIV-1 virus-host protein interactions using virus and host sequence motifs. BMC Med Genomics. 2009;2:27.
7. Hagai T, Azia A, Babu MM, Andino R. Use of host-like peptide motifs in viral proteins is a prevalent strategy in host-virus interactions. Cell Rep. 2014 Jun;7(5):1729–1739.
8. Fu W, Sanders-Beer BE, Katz KS, Maglott DR, Pruitt KD, Ptak RG. Human immunodeficiency virus type 1, human protein interaction database at NCBI. Nucleic Acids Res. 2009 Jan;37(Database issue):D417–422.
9. Bateman A, Martin MJ, O'Donovan C, Magrane M, Apweiler R, Alpi E, et al. UniProt: a hub for protein information. Nucleic Acids Res. 2015 Jan;43(Database issue):D204–212.
10. Sarkar D, Jana T, Saha S. LMPID: a manually curated database of linear motifs mediating protein-protein interactions. Database (Oxford). 2015;2015.
